# Development and validation of a clinical predictive model for the risk of recurrence after unilateral biportal endoscopic diskectomy

**DOI:** 10.3389/fsurg.2026.1778866

**Published:** 2026-07-27

**Authors:** Zhen Yi, Changhe Liao, Ting Wu, Jinping Liu, Kunhua Zeng, Xian He

**Affiliations:** 1 Department of Traditional Chinese Medicine, Foshan No. 5 People's Hospital, Foshan, Guangdong; 2 Guangzhou Panyu District Traditional Chinese Medicine Hospital, Orthopaedic Department, Guangzhou, Guangdong; 3 Guangzhou University of Chinese Medicine Graduate School, Guangzhou, Guangdong

**Keywords:** decision curve analysis, nomogram, nomogram prediction model, postoperative recurrence, risk factors, UBED

## Abstract

**Background:**

To identify the independent risk factors for postoperative recurrence following unilateral biportal endoscopic discectomy (UBED) for lumbar disc herniation (LDH), and to develop and validate a recurrence risk prediction model.

**Objective:**

To analyze the risk factors contributing to postoperative recurrence in patients with LDH treated by UBED, and to construct and validate a predictive nomogram for recurrence risk after UBED.

**Methods:**

A total of 257 patients with single-level LDH who underwent UBED were retrospectively enrolled, among whom 24 presented postoperative recurrence, corresponding to a recurrence rate of 9.34%. The cohort was split into a training set (*n* = 179) and a validation set (*n* = 78) at a 7:3 ratio. Thirty clinical and radiological parameters were collected. Least Absolute Shrinkage and Selection Operator (LASSO) regression combined with multivariate logistic regression was applied to screen independent risk factors, and a nomogram prediction model was subsequently established. Model performance was assessed via receiver operating characteristic (ROC) curves, Hosmer-Lemeshow (H-L) test, calibration curves, and decision curve analysis (DCA).

**Results:**

Twelve independent risk factors for postoperative recurrence were identified. The area under the ROC curve (AUC) reached 0.976 in the training set and 0.953 in the validation set. The P values of the H-L test were both >0.05 in the two datasets, indicating favorable model calibration. The model yielded significant clinical net benefit within threshold probabilities of 0–0.92 (training set) and 0–0.89 (validation set).

**Conclusions:**

The established nomogram incorporating 12 clinical and radiological variables exhibits satisfactory discrimination and calibration for predicting recurrence after UBED. It serves as a quantitative tool for rapid identification of patients at high recurrence risk in clinical practice. Nevertheless, limitations including single-center retrospective design, relatively small sample size, and an events-per-variable (EPV) ratio of approximately 2:1 (far below the recommended threshold) introduce potential overfitting and limited generalizability. This model is only applicable for preliminary screening of high-risk individuals and preoperative patient counseling, rather than acting as the sole basis for clinical decision-making. Multicenter, large-sample prospective cohort studies are warranted for further external validation and model refinement in the future.

## 1 Introduction

Lumbar stability is the core foundation for maintaining normal physiological function of the spine and ensuring trunk mobility, which relies on the synergistic action of multiple structures including bones, intervertebral discs, facet joints, and paraspinal muscles. The lumbar multifidus muscle (LMM), as the deepest core stabilizing muscle group of the spine, is short and segmentally distributed, tightly connecting adjacent vertebral segments, and plays an irreplaceable role in regulating lumbar biomechanics. This muscle group is primarily responsible for maintaining fine lumbar posture and controlling segmental motion. It can effectively resist abnormal stresses such as spinal rotation and shear, constrain micro-displacement of adjacent vertebral bodies, and prevent the intervertebral disc from bearing unbalanced and overloaded mechanical stimulation, making it a key anatomical structure for maintaining lumbar segmental stability (1).

In addition to paraspinal muscles, structures such as the lumbar intervertebral disc, endplates, and facet joints also participate in the regulation of lumbar homeostasis. Anatomical and radiological features including lumbar facet orientation (FO), facet tropism (FT), and the degree of adjacent segment disc degeneration are closely associated with lumbar mechanical distribution, disease occurrence, and prognosis, providing important anatomical evidence for the onset, progression, and postoperative outcomes of lumbar disc-related diseases.

Lumbar disc herniation (LDH) is a highly prevalent spinal disease caused by rupture of the annulus fibrosus, herniation of the nucleus pulposus, and degenerative changes of the intervertebral disc, which subsequently compresses and irritates nerve roots. It is also the leading cause of chronic low back pain, lower limb numbness, and impaired limb motor function in clinical practice. Epidemiological data show that the global incidence of isolated LDH ranges from 13% to 31%, and the overall incidence of various lumbar disc lesions combined with radicular neurological symptoms can reach 12% to 40%. The extremely high prevalence not only severely impairs patients’ quality of life and limits daily activity, but also imposes a heavy disease burden on patients’ families and the global public health system (2–4).

Clinical evidence confirms that the onset and symptom progression of LDH often coexist with multiple lumbar degenerative conditions. Among them, lumbar endplate inflammation (Modic changes), lumbar spinal stenosis, intervertebral disc degeneration (IVDD), and fat infiltration of paraspinal muscles are core risk factors that induce and aggravate low back pain. Currently, clinical management of LDH is divided into conservative treatment and surgical intervention. Conservative treatment is indicated for patients with mild symptoms, but for patients with moderate to severe disease who fail conservative therapy, experience recurrent symptoms, or present with progressive neurological impairment, surgical intervention is the core treatment to relieve nerve compression and restore lumbar function.

Notably, postoperative recurrence of LDH remains a key challenge affecting clinical efficacy. Existing studies indicate that the overall postoperative recurrence rate of LDH can reach 7.5%–18.1% (5). Patients with recurrence redevelop typical symptoms such as lumbocrural pain and lower limb numbness. Some patients respond poorly to conservative treatment and require revision surgery, which greatly increases physical and psychological trauma as well as medical economic burden for patients.

With the rapid advancement of minimally invasive spine surgery, traditional open lumbar surgery has been gradually replaced by minimally invasive endoscopic techniques due to its drawbacks such as large trauma, substantial bleeding, slow postoperative recovery, and significant paraspinal muscle injury. Percutaneous endoscopic lumbar discectomy has become the mainstream minimally invasive procedure for LDH owing to its remarkable advantages of minimal invasiveness, safety, and rapid postoperative rehabilitation.

Unilateral biportal endoscopic discectomy (UBED) is an emerging improved minimally invasive spinal endoscopic technique. By establishing separate observation and working channels, it provides a clear surgical field and flexible instrument manipulation. It can efficiently manage complex cases that are difficult to address with traditional endoscopic surgery, such as complex disc herniation, disc calcification, and concomitant lumbar spinal stenosis. With wider clinical applicability and superior safety and efficacy, UBED has been rapidly popularized worldwide and has gradually become a core procedure for minimally invasive treatment of LDH, regarded as an important development direction of minimally invasive spine surgery.

Compared with traditional open surgery and conventional single-channel endoscopic surgery, UBED has prominent advantages in preserving normal lumbar anatomy, reducing paraspinal muscle dissection injury, and maintaining postoperative lumbar stability, with overall clinical efficacy significantly better than traditional procedures (6). Meanwhile, clinical data confirm that the overall recurrence rate after UBED is lower than that after traditional open surgery. However, recurrent lumbar disc herniation (RLDH) remains an unavoidable core complication after UBED, which directly restricts long-term surgical outcomes (7).

To date, domestic and international studies on risk factors for postoperative recurrence of lumbar disc have made certain progress. Existing evidence confirms that age, sex, smoking history, diabetes, body mass index (BMI), occupational factors, type of disc herniation, degeneration grade, disc height index (DHI), sagittal range of motion (sROM), and thoroughness of nucleus pulposus removal are major risk factors for recurrence after conventional endoscopic, microscopic, and open discectomy (8, 9). Nevertheless, current studies still have notable shortcomings and gaps, which are insufficient to support precise clinical risk prevention and control for UBED.

First, most existing studies on recurrence risk factors focus on traditional procedures or conventional single-channel endoscopic surgery. Specific studies on radiological risk factors for recurrence after UBED are relatively scarce. In particular, the association and predictive value of key radiological indicators such as lumbar facet orientation, facet tropism, and adjacent segment disc degeneration with recurrence after UBED have not been systematically verified, and clinical evidence is lacking.

Second, current studies on recurrence after UBED are mostly univariate analyses, lacking a systematic and multidimensional integrated assessment system for risk factors. Conclusions from different studies are controversial and inconsistent, making it impossible to comprehensively and accurately identify populations at high risk of postoperative recurrence. Most critically, there is currently no mature and reliable prediction model for recurrence risk after UBED in clinical practice. Relevant research is still in its infancy, and there is no standardized prediction tool to guide preoperative risk assessment, surgical plan optimization, and postoperative individualized intervention (10–12). As a result, clinicians cannot identify high-risk patients in advance and implement targeted preventive measures, making it difficult to effectively reduce the postoperative recurrence rate.

Given the current clinical research status and existing gaps, it is of great clinical necessity and research value to systematically explore and verify independent risk factors for recurrence after UBED and to construct a scientific, accurate, and practical prediction model for postoperative recurrence risk. This study aims to systematically screen potential risk factors for recurrence after UBED through retrospective clinical data analysis, identify core independent influencing factors, and further construct and validate a dedicated clinical prediction model. The findings can fill the current research gap in this field, provide objective and scientific reference for clinicians to evaluate patients’ recurrence risk preoperatively, formulate individualized surgical strategies, and implement precise postoperative interventions. It will help realize refined diagnosis and treatment of LDH after minimally invasive surgery, and has important theoretical significance and clinical application prospects for reducing recurrence rate after UBED, improving long-term prognosis, and enhancing clinical care quality.

## 2 Data and methods

### 2.1 Study population

This study retrospectively included patients with single-segment lumbar disc herniation who underwent UBED surgery in the Department of Orthopedics, Panyu District Hospital of Traditional Chinese Medicine, from February 2021 to March 2025. Data were collected by two orthopedic residents under the guidance of an associate chief physician from the hospital's electronic medical record system, and discrepancies were resolved through third-party arbitration. Variables with a missing value rate exceeding 10% were excluded, and the remaining missing data were processed using multiple imputation.

### 2.2 Inclusion criteria

(1) Patients who underwent UBED as the surgical treatment, performed by spine surgeons with UBED qualifications and experience; (2) Patients with a preoperative diagnosis of single-segment lumbar disc herniation, confirmed by preoperative lumbar MRI or CT showing single-segment (e.g., L4/5, L5/S1) disc herniation, with imaging findings consistent with clinical symptoms and signs. Typical clinical symptoms include low back pain with unilateral lower limb radiating pain, numbness or weakness, positive straight leg raise test, etc., or failure of systematic conservative treatment for more than 6 weeks; (3) Patients whose clinical symptoms, signs, and imaging findings are consistent with LDH, or those with cauda equina syndrome, or recurrent disc herniation after previous surgery; (4) Follow-up time of more than 3 months, with complete and accessible postoperative clinical and imaging follow-up data; (5) All surgeries were performed through the multifidus triangle and the muscle-lamina gap approach; (6) Key baseline data required for the study were available, baseline imaging data were complete with standardized scanning parameters (e.g., uniform magnetic field strength, slice thickness) to reduce measurement errors, and intraclass correlation coefficients (ICC≥0.8) were reported to assess inter-observer agreement; (7) Postoperative recurrence after UBED was defined as: recurrence of radicular symptoms such as low back and leg pain, lower limb radiating pain, numbness or weakness consistent with preoperative symptoms at the same surgical segment after ≥3 months of follow-up; and lumbar MRI (1.5 T) confirmed re-herniation of the disc at the same segment or re-herniation of residual nucleus pulposus tissue compressing the corresponding nerve root.

### 2.3 Exclusion criteria

(1) Patients aged <18 years, or with neuropsychiatric disorders unable to complete relevant scales; (2) Patients with incomplete postoperative follow-up data or lost to follow-up; patients with previous lumbar disc surgery or preoperative history of disc herniation recurrence; (3) Patients with concomitant severe lumbar instability, spondylolisthesis (grade II or above), spinal deformity, spinal tumor, infection, fracture, or other spinal diseases requiring simultaneous or staged treatment at the surgical site; (4) Complicated with severe cardiac, cerebral, pulmonary, or renal underlying diseases; (5) Imaging evidence of lumbar instability; (6) Patients with multi-level lumbar disc herniation who underwent simultaneous UBE surgery; (7) Patients with surgical failure or unrelieved symptoms within a short period (<3 months) due to definite causes (e.g., hematoma, infection, positioning error). Evaluators (radiologists and statisticians) were blinded to patient grouping to avoid measurement bias. If grouping information was identifiable in retrospective data, data were re-coded by an independent third-party team. Imaging parameters were independently measured by two spine surgeons and re-evaluated one week apart to assess measurement reliability. The final result was the average of the two measurements. This study was conducted in accordance with the Declaration of Helsinki and approved by the Ethics Committee of Guangzhou Panyu District Hospital of Traditional Chinese Medicine (approval No. PZY-LL-KY-2025089).

### 2.4 Methodology

Given the low surgical volume and incidence of recurrence for UBED, this study adopted a retrospective matched case-control design to investigate multiple potential exposure factors for LDH recurrence after UBED. Clinical data were collected from the hospital medical record system. The following imaging parameters were compared between the two groups: type of disc herniation, Modic changes and modified Pfirrmann grade of disc degeneration at the surgical level, disc height index (DHI), sagittal range of motion (sROM), nucleus pulposus herniation area at the surgical level [herniated volume = (slice spacing + slice thickness) × *Σ* area of herniation per layer, mm^2^], lumbar facet orientation (FO) and facet tropism (FT), size of postoperative annulus fibrosus tear, cross-sectional area of intervertebral foramen (CSAF), spondylolisthesis index [posterior displacement of upper vertebra relative to lower vertebra (A)/width of upper vertebra (B)], LMM cross-sectional area, LMM fat ratio (fat ratio = LMM fat area/LMM cross-sectional area), and completeness of nucleus pulposus removal. Data were collected by two professional orthopedic researchers using the electronic medical record system of Guangzhou Panyu District Hospital of Traditional Chinese Medicine. Data entry was performed in a blinded manner and double-checked to ensure authenticity and accuracy. Missing data processing: Four variables [preoperative leg pain Visual Analog Scale (VAS) score, modified Pfirrmann grade, sROM, and postoperative trauma history] had a missing rate >10% and were excluded. The remaining variables were processed using MICE (multivariate imputation by chained equations) to generate 5 imputed datasets, with 20 iterations until convergence. Results were pooled using Rubin's rules. Imputation methods: predictive mean matching (PMM) for continuous variables, logistic regression for binary variables, and multinomial logistic regression for ordinal/multinomial variables. Supplementary Table S1 in the Supplementary Material compares baseline characteristics between the complete-case dataset and imputed dataset to verify imputation validity. General characteristics of included patients included sex, age, body weight, alcohol consumption, smoking, hypertension, diabetes, coronary heart disease, hyperlipidemia, surgical level, preoperative low back pain VAS score, preoperative Oswestry Disability Index (ODI), symptom duration, high-intensity physical labor, and completeness of nucleus pulposus removal. On sagittal T2-weighted images, an annulus fibrosus tear was defined as a focal hyperintense linear or fissure-like interruption at the posterior/posterolateral disc margin, connecting the nucleus pulposus cavity to the spinal canal or intervertebral foramen. Only clear transmural tears visible on the first postoperative MRI were measured, excluding hematoma or postoperative inflammatory changes. Measurement method: two independent spine surgeons used the electronic caliper built into the Picture Archiving and Communication System (PACS) to measure the maximum anteroposterior diameter (mm) on sagittal views and the maximum transverse diameter (mm) on axial views. The geometric mean of the two values was defined as “tear size”. Each surgeon performed two measurements (2 weeks apart) and the average value was used.

### 2.5 UBED surgical procedure

Routine bilateral ligamentum flavum resection and lateral recess decompression were performed in all UBED procedures at our center. Surgery was performed under general anesthesia with the patient in the prone position. C-arm fluoroscopy was used to localize the surgical level. A horizontal line was drawn through the midpoint of the spinous process of the upper vertebra of the target level, intersecting with the line connecting the medial borders of the pedicles. The observation and working portals were marked 1.5 cm above and below the intersection point, respectively. A dilator was used to sequentially separate paraspinal muscles and soft tissues. The spinal endoscope was inserted through the upper portal, and the working cannula was placed through the lower portal. Correct positioning at the target intervertebral space was confirmed by C-arm fluoroscopy. An endoscopic radiofrequency probe was used to clear soft tissue over the lamina space to expose the upper and lower laminae. A Kerrison rongeur and high-speed drill were used to resect the inferior edge of the lamina and the medial facet joint. Bone was drilled upward from the inferior margin of the ipsilateral lamina to the attachment of the ligamentum flavum. The ligamentum flavum and contralateral recess were addressed using a nerve dissector, Kerrison rongeur, and drill. After identifying the nerve root at the surgical level, the herniated nucleus pulposus was removed with pituitary rongeurs. Radiofrequency ablation was applied to the remaining nucleus pulposus. The surgical level was re-inspected, the endoscope was removed, the wound was irrigated and sutured, and a sterile dressing was applied. Postoperative routine care, 24 hours of bed rest, and waist brace immobilization for at least 3 weeks were prescribed (13). Two researchers not involved in the surgery independently reviewed surgical records based on unified criteria (e.g., explicit documentation of “definite massive residual” rather than “small amount of fragmented tissue”). Data were included only when both reviewers agreed.

### 2.6 Imaging manifestations of predictive models for postoperative recurrence of UBED

**Figure 1:**
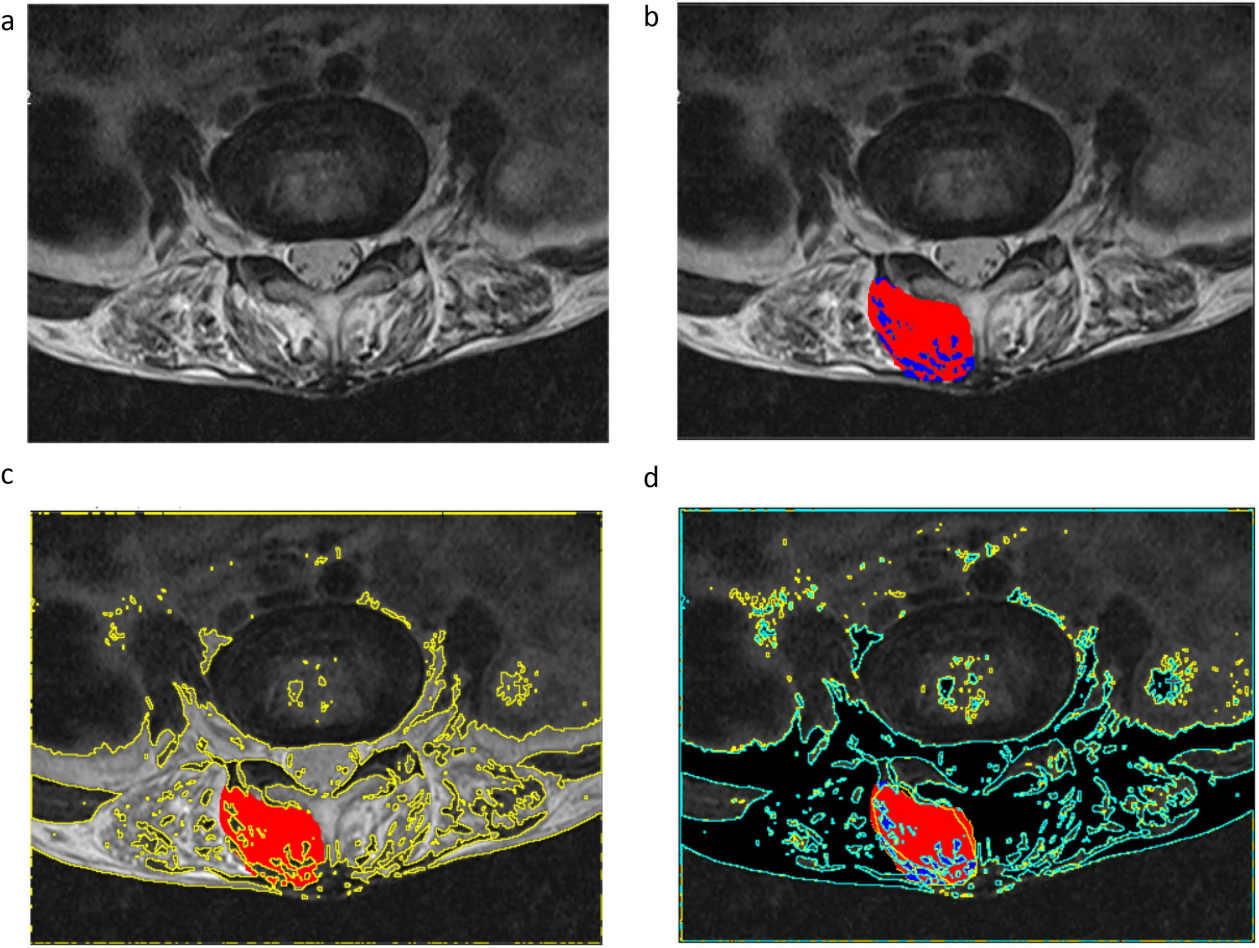
All images (including Figure 1a) are of the lumbar MRI scans of a patient at our hospital prior to treatment. ImageJ software was used to measure the lumbar multifidus muscle (LMM) in the corresponding segment. The blue and red areas marked in Figure 1b represent the LMM, while the white and dark blue areas in Figures 1c and 1d represent fat.

Preoperative and postoperative lumbar MRI (Siemens Magnetom Espree 1.5 T, Germany), lumbar digital radiography (Siemens Ysio system), and CT (Philips Ingenuity 64-slice CT) images were retrieved from the hospital electronic medical record system. ImageJ (Media Cybernetics, Bethesda, MD, USA) was used to measure LMM cross-sectional area and quantify LMM fat infiltration. Fat infiltration of paraspinal muscles was graded using the modified Goutallier classification: Grade 0: Normal muscle; Grade I: Minimal fatty streaks within muscle; Grade II: Fat infiltration up to 25%; Grade III: Fat infiltration between 25% and 50%; Grade IV: Fat infiltration exceeding 50% (See Figure 1).

### 2.7 Main outcome measures

LASSO regression and multivariate logistic regression were used to screen independent risk factors for postoperative recurrence after UBED. R software was used to construct the nomogram model and evaluate the model performance. The area under the ROC curve (AUC) was used to verify the predictive results and clinical utility. The AUC value ranges from 0.5 to 1, with a larger value closer to 1 indicating better diagnostic or predictive performance. The “rms” package in R was used to draw calibration curves (1000 times Bootstrap resampling) and perform the Hosmer-Lemeshow goodness-of-fit test to assess the calibration of the model. Decision curve analysis (DCA) was used to evaluate the potential clinical application value of the nomogram. The forestmodel package was used to create a forest plot to visually present the results of multivariate logistic regression analysis.

### 2.8 Statistical analysis

Statistical analysis was performed using SPSS 27.0 and R version 4.3.2. Patients were randomly divided into a training cohort and a validation cohort at a 7:3 ratio using the sample function with a fixed random seed. The training cohort was used for model construction, and the validation cohort for model validation. Baseline balance between groups was tested. Normally distributed continuous variables are expressed as mean ± standard deviation (x̅ ± s), and compared between groups using the independent samples t-test. Non-normally distributed continuous variables are expressed as median (interquartile range) [M (QR)], and compared using the Mann–Whitney U test. Categorical variables are expressed as frequencies and proportions, and compared using the chi-square test. A P-value < 0.05 was considered statistically significant. LASSO regression was first used for preliminary variable screening. Combined with clinical relevance, variables were selected for inclusion in multivariate logistic regression to construct the nomogram model. Bootstrap resampling was used for internal validation. Model discrimination, calibration, and clinical benefit were evaluated using corrected AUC, Hosmer-Lemeshow test, and DCA. The optimal *λ* value for LASSO was determined by 10-fold cross-validation.

## 3 Results

### 3.1 Participant number analysis

A total of 280 LDH patients who underwent UBED were initially screened. After excluding 23 patients who did not meet inclusion criteria, 257 patients were finally included, with a follow-up duration of at least 3 months. Twenty-four patients experienced recurrence, giving a recurrence rate of 9.34%, consistent with the reported range of 7.5%–18.1% for UBED in the literature. The events-per-variable (EPV) ratio was approximately 2:1, far below the recommended threshold of EPV ≥ 10:1 for clinical prediction models, indicating a notable risk of overfitting. The 7:3 split yielded 179 patients in the training cohort and 78 in the validation cohort. The validation cohort contained only approximately 7 recurrence events, which was insufficient to robustly validate a model with 12 predictors.

### 3.2 Comparison of clinical characteristics between training and validation groups

**Figure 2:**
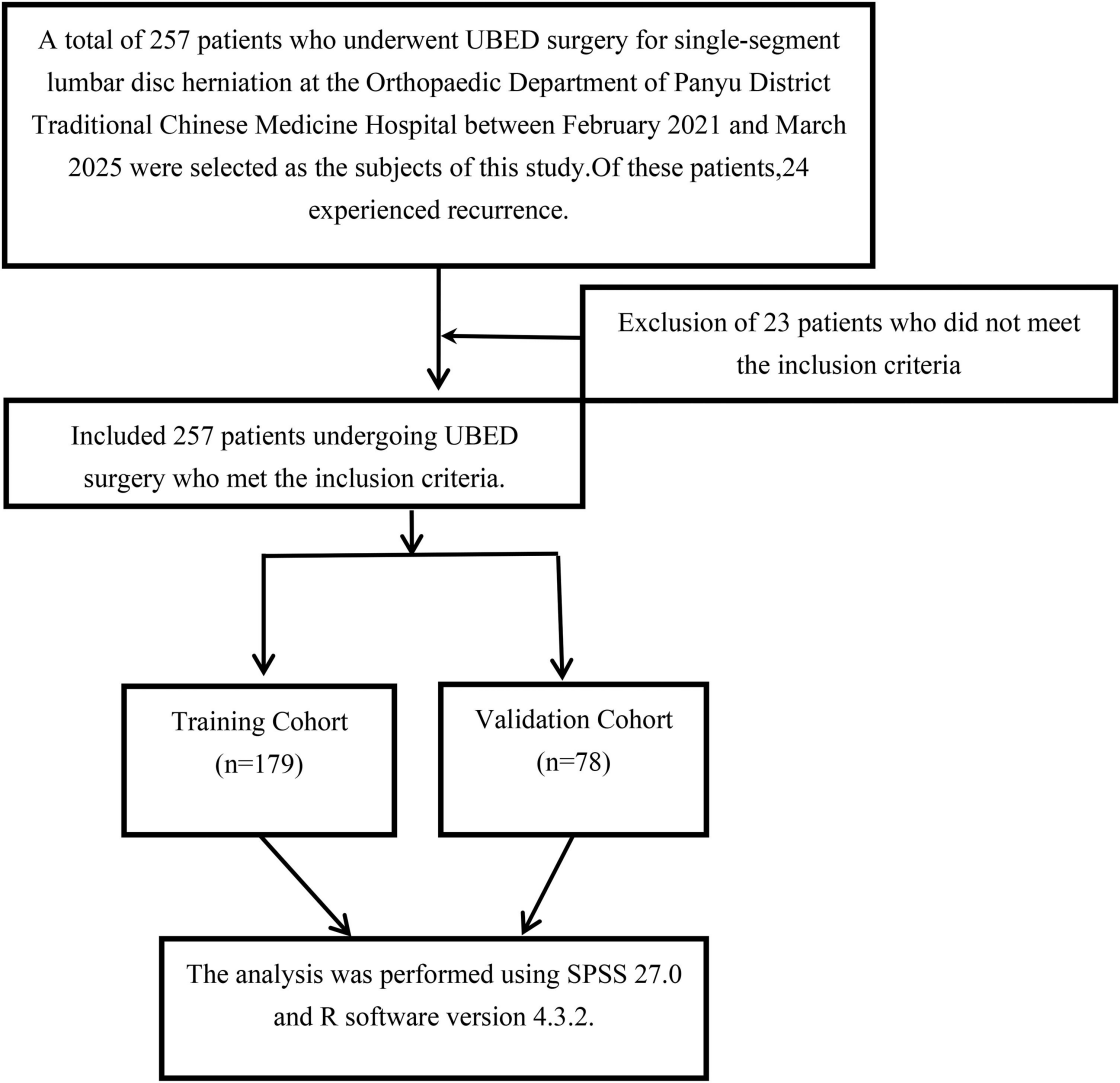
Flow chart depiction of the study protocol.

**Figure 3:**
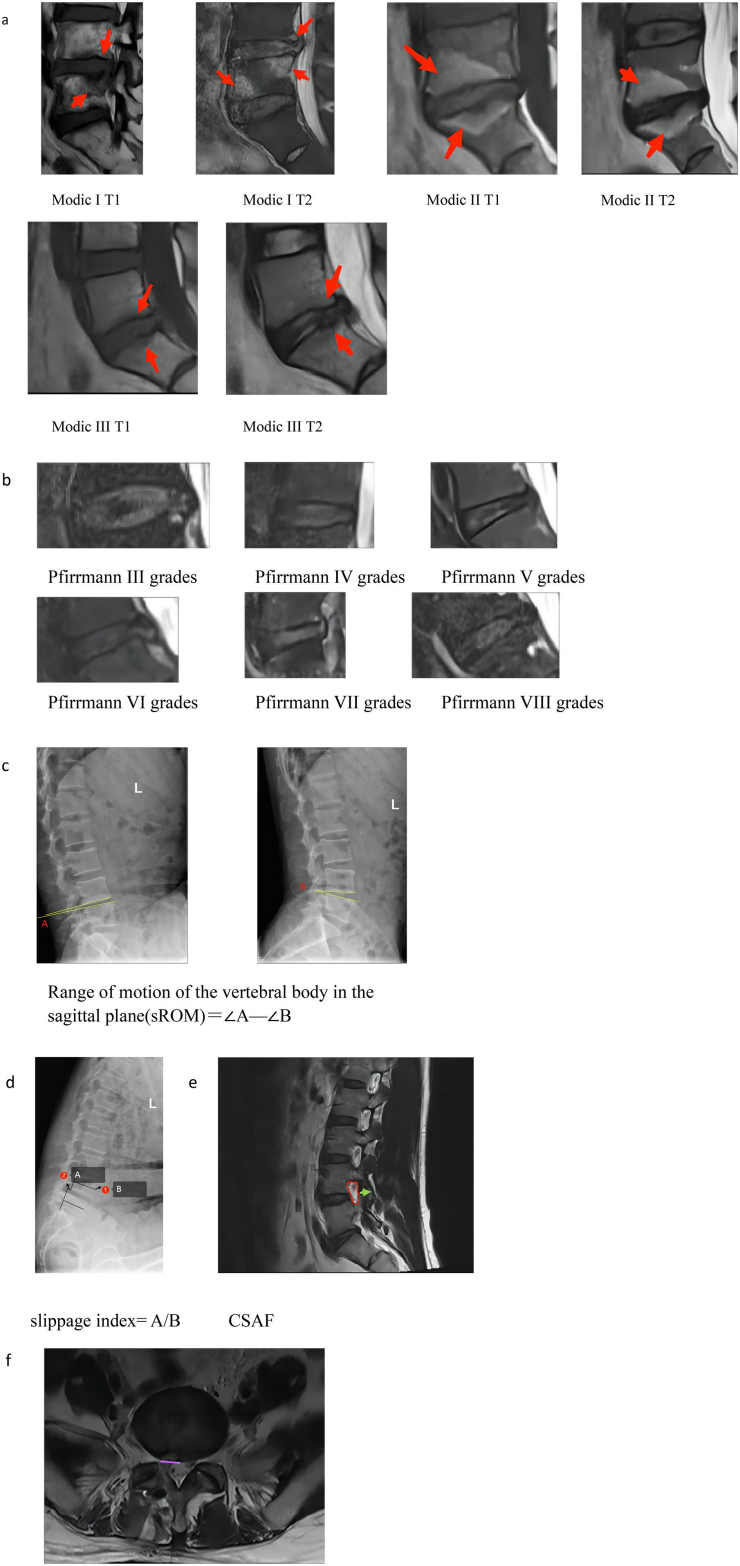
**(a)** modic changes in the surgical segment; **(b)** modified pfirrmann grading of the surgical segment; **(c)** sagittal plane range of motion (sROM); **(d)** slippage index measurement:measurement of the posterior displacement distance **(A)** and width **(B)** of the upper vertebra relative to the lower vertebra; **(e)** the area enclosed by the red lines represents the cross-sectional area of the intervertebral foramen (CSAF); **(f)** the purple lines represent the size of the annular tear postoperatively.

**Figure 4:**
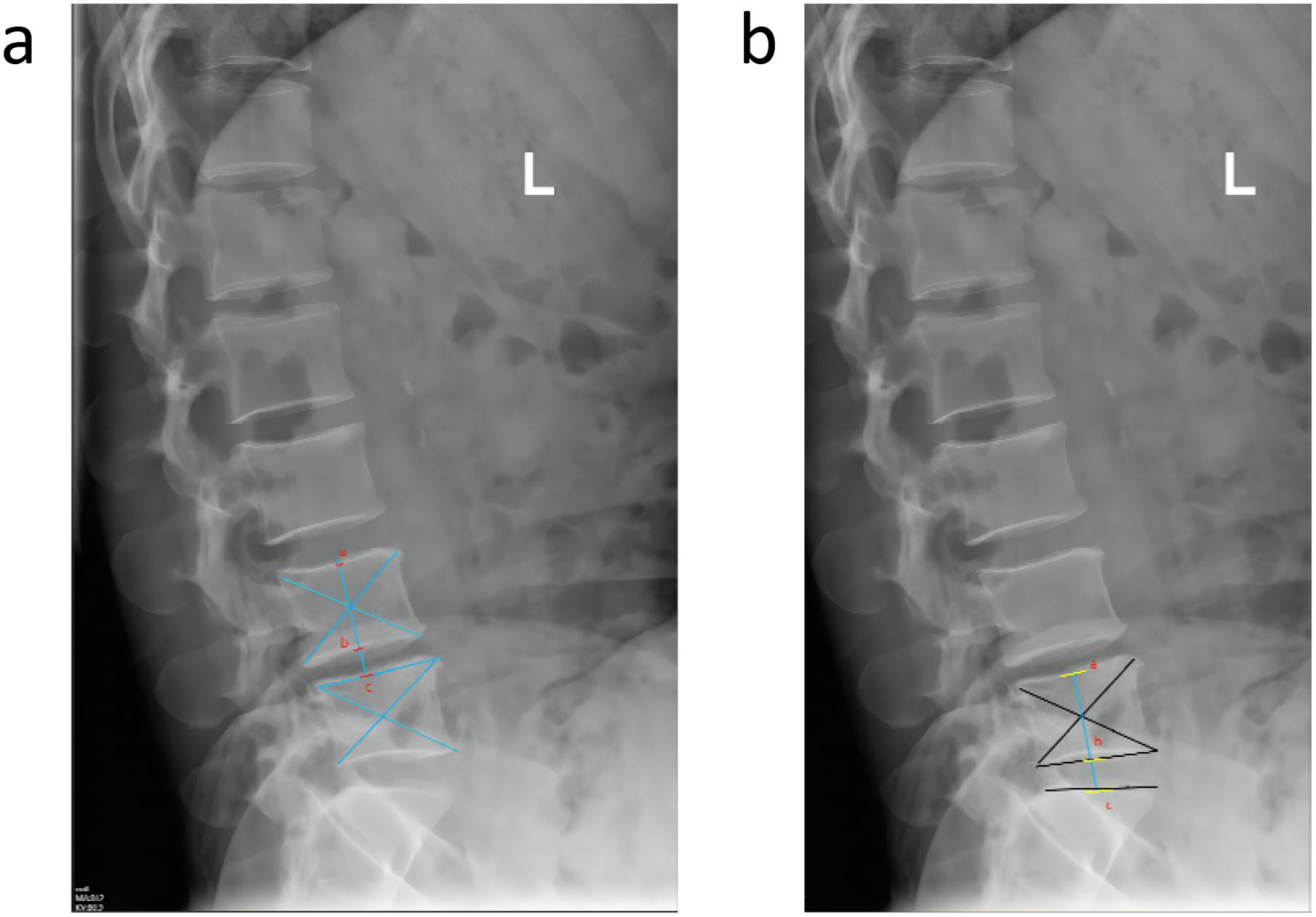
Calculate the disc height Index (DHI): **(a)** L4-L5 = bc/ab (blue lines connecting red markers); **(b)** L5-S1 = ef/de.

Statistically significant differences were observed between the two groups in BMI, smoking, diabetes, Modic changes, surgical-level Modic changes, DHI, FO, FT, type of disc herniation, nucleus pulposus herniation area, lumbar spondylolisthesis index, preoperative low back pain VAS, preoperative leg pain VAS, preoperative ODI, postoperative annulus fibrosus tear size, CSAF, LMM cross-sectional area, LMM fat ratio, and incomplete nucleus pulposus removal (all P < 0.05). No significant differences were found in age, sex, alcohol consumption, hypertension, coronary heart disease, hyperlipidemia, surgical level, modified Pfirrmann grade, sROM, symptom duration, high-intensity physical labor, or postoperative trauma history (all *P* > 0.05). Detailed results are shown in Table 1, Figures 2–4).

**Table 1:** Comparison of general data between the two groups of patients.

Variables	Training Group(*n* = 179)	Verification Group(*n* = 78)	Statistical Value	P-value
Age($\bar{x}$ ± s，years):	51.74 ± 14.62	53.42 ± 14.34	Z = −0.931	0.352
Sex:			χ^2^ = 1.102	0.294
Male	97 (54.2%)	36 (46.2%)		
Female	82 (45.8%)	42 (53.8%)		
BMI($\bar{x}$ ± s，kg/m^2^):			χ^2^ = 10.36	0.006
＜24	72 (40.22%)	19 (24.36%)		
24∼27	68 (37.99%)	28 (35.90%)		
≥27	39 (21.79%)	31 (39.74%)		
Smoke{[M(QR)]}:			χ^2^ = 15.093	<0.001
Yes	49 (27.4%)	4 (5.1%)		
No	130 (72.6%)	74 (94.9%)		
Drinking{[M(QR)]}:			χ^2^ = 0.142	0.706
Yes	75 (41.9%)	30 (38.5%)		
No	104 (58.1%)	48 (61.5%)		
Hypertension{[M(QR)]}:			χ^2^ = 0.006	0.938
Yes	53 (29.6%)	22 (28.2%)		
No	126 (74.4%)	56 (71.8%)		
Diabetes{[M(QR)]}:			χ^2^ = 25.371	<0.001
Yes	9 (5.0%)	22 (28.2%)		
No	170 (95.0%)	56 (71.8%)		
Coronary Heart Disease{[M(QR)]}:			χ^2^ = 2.659	0.103
Yes	29 (16.2%)	6 (7.7%)		
No	150 (83.8%)	72 (92.3%)		
Hyperlipidaemia{[M(QR)]}:			χ^2^ = 0.449	0.503
Yes	55 (30.7%)	28 (35.9%)		
No	124 (69.3%)	50 (64.1%)		
Surgical segment{[M(QR)]}:			χ^2^ = 6.393	0.094
L5/S1	70 (39.1%)	24 (30.8%)		
L4/L5	90 (50.3%)	51 (65.4%)		
L3/L4	17 (9.5%)	3 (3.8%)		
L2/3	2 (1.1%)	0 (0)		
Modic changes in the surgical segment{[M(QR)]}:			χ^2^ = 7.701	0.021
Type I	126 (70.4%)	46 (59.0%)		
Type II	15 (8.4%)	16 (20.5%)		
Type III	38 (21.2%)	16 (20.5%)		
Surgical segment modified Pfirrmann grading{[M(QR)]}:			χ^2^ = 9.271	0.099
Grade III	22 (12.3%)	8 (10.2%)		
Grade IV	16 (9.0%)	11 (14.1%)		
Grade V	40 (22.3%)	8 (10.2%)		
Grade VI	41 (22.9%)	14 (18.0%)		
Grade VII	20 (11.2%)	13 (16.7%)		
Grade VIII	40 (22.3%)	24 (30.8%)		
DHI($\bar{x}$ ± s):			χ^2^ = 7.98	0.018
＜0.25	34 (18.99%)	5 (6.41%)		
0.25∼0.35	83 (46.37%)	36 (46.15%)		
≥0.35	62 (34.64%)	37 (47.44%)		
sROM($\bar{x}$ ± s):	5.43 ± 4.11	4.95 ± 3.80	Z = −0.810	0.418
FO($\bar{x}$ ± s):	34.82 ± 10.46	40.68 ± 12.10	T = 3.940	<0.001
FT($\bar{x}$ ± s):	5.40 ± 3.17	3.60 ± 2.75	Z = −4.437	<0.001
Types of herniated discs{[M(QR)]}:			χ^2^ = 27.532	<0.001
Central Type	59 (33.0%)	38 (48.7%)		
Paracentral Type	86 (48.0%)	22 (28.2%)		
Sequestration Type	14 (7.8%)	18 (23.1%)		
Extrusion Type	20 (11.2%)	0 (0)		
Area of disc protrusion ($\bar{x}$ ± s,mm^2^):	114.13 ± 44.69	101.75 ± 69.54	Z = −3.085	0.002
Lumbar Spondylolisthesis Index Measurement ($\bar{x}$ ± s):	0.13 ± 0.06	0.08 ± 0.05	Z = −6.030	<0.001
Preoperative low back pain VAS($\bar{x}$ ± s):			χ^2^ = 80.36	<0.001
<7	112 (62.57%)	1 (1.28%)		
≥7	67 (37.43%)	77 (98.72%)		
Preoperative leg pain VAS ($\bar{x}$ ± s):			χ^2^ = 70.56	<0.001
<7	122 (68.16%)	8 (10.26%)		
≥7	57 (31.84%)	70 (89.74%)		
Preoperative ODI($\bar{x}$ ± s)：			χ^2^ = 63.17	<0.001
≤40	6 (3.35%)	10 (12.82%)		
40∼60	59 (32.96%)	60 (76.92%)		
≥61	114 (63.69%)	8 (10.26%)		
Duration of symptoms ($\bar{x}$ ± s,days):	1050.27 ± 1250.43	1092.66 ± 1313.20	Z = −1.387	0.165
Heavy Physical Labour{[M(QR)]}:			χ^2^ = 0.625	0.429
Yes	67 (37.4%)	34 (43.6%)		
No	112 (62.6%)	44 (56.4%)		
History of Post-Operative Trauma{[M(QR)]}:			χ^2^ = 0.102	0.749
Yes	68 (38.0%)	32 (41.0%)		
No	111 (62.0%)	46 (59.0%)		
Size of post-operative annular tear{[M(QR)],mm}:	14.81 ± 1.57	14.23 ± 1.66	T = −2.702	0.007
Transverse area of the intervertebral foramen(x ± s,mm^2^):	111.97 ± 11.89	115.69 ± 11.41	T = 2.338	0.02
LMM cross-sectional area(x ± s,mm^2^):	1537.41 ± 130.43	1633.42 ± 139.59	T = 5.310	<0.001
LMM fat ratio(%):	33.73 ± 8.67	40.51 ± 7.89	T = 5.917	<0.001
Incomplete removal of the nucleus pulposus{[M(QR)]}:			χ^2^ = 12.521	<0.001
Yes	36(20.1%)	33(42.3%)		
No	143(79.9%)	45(57.7%)		

### 3.3 Variable coding for recurrence analysis

Thirty variables were included in the analysis. Selected variables were coded as follows: quantitative variables were grouped per clinical criteria, categorical variables were factorized, and string variables were digitized. Continuous variables (BMI, DHI, preoperative low back pain VAS, preoperative ODI, LMM cross-sectional area, LMM fat ratio) were entered directly into regression models. For baseline presentation, they were grouped by clinical cutoffs: BMI (<24, 24–27, ≥27), preoperative low back pain VAS (<7, ≥7), preoperative leg pain VAS (<7, ≥7), preoperative ODI (≤40, 41–60, ≥61), DHI (<0.25, 0.25–0.35, ≥0.35) (14–17). Coding rules: Sex: male = 1, female = 2; Alcohol consumption, smoking, hypertension, diabetes, coronary heart disease, hyperlipidemia: yes = 1, no = 0; Surgical level: L5/S1 = 1, L4/L5 = 2, L3/L4 = 3; High-intensity physical labor, postoperative trauma history: yes = 1, no = 0；Type of disc herniation: central = 1, paracentral = 2,sequestered = 3,extruded = 4；Surgical-level Modic changes: type I = 1,type II = 2,type III = 3；Modified Pfirrmann grade: grade I = 1, grade II = 2, grade III = 3, grade IV = 4, grade V = 5,grade VI = 6, grade VII = 7, grade VIII = 8; Incomplete nucleus pulposus removal: yes = 1,no = 0; No statistically significant difference in baseline distribution was found between the training and validation groups (P > 0.05),as shown in Table 1.

### 3.4 Variable selection for recurrence prediction

**Figure 5:**
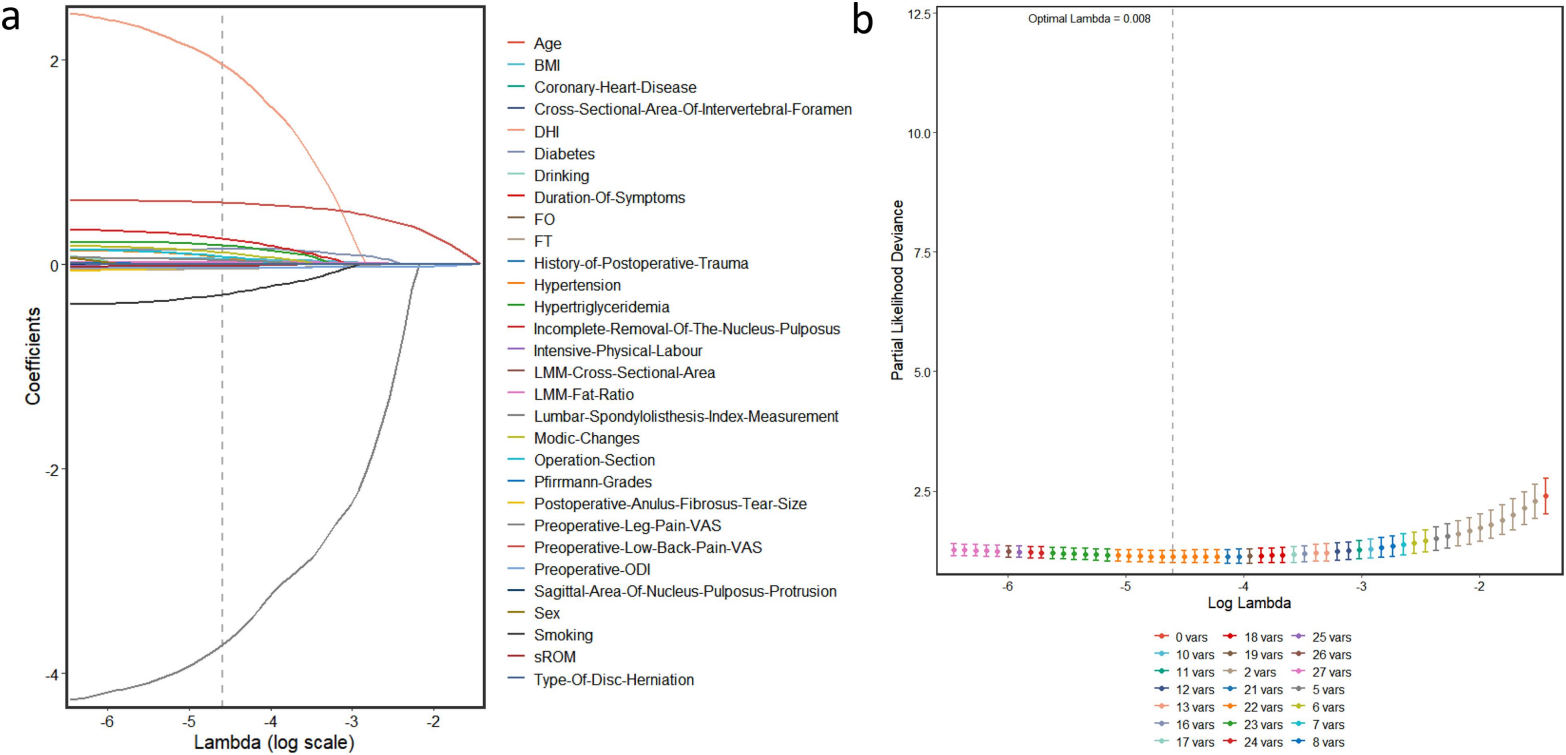
LASSO regression model screening of predictive variables.

The glmnet package was used to fit a LASSO regression model for variable selection from 30 candidate variables. Variables with non-zero coefficients were retained as important features. As the penalty parameter *λ* increases, variable coefficients shrink progressively toward zero. Using 10-fold cross-validation, the optimal *λ* value was determined as 0.01008781.LASSO regression identified 18 characteristic variables associated with recurrence after UBED:BMI, smoking, diabetes, Modic changes, surgical-level Modic changes, DHI, FO, FT, type of disc herniation, nucleus pulposus herniation area, lumbar spondylolisthesis index, preoperative low back pain VAS, preoperative leg pain VAS, preoperative ODI, postoperative annulus fibrosus tear size, CSAF, LMM cross-sectional area, LMM fat ratio, and incomplete nucleus pulposus removal (Figure 5).

### 3.5 Model construction and validation

**Figure 6:**
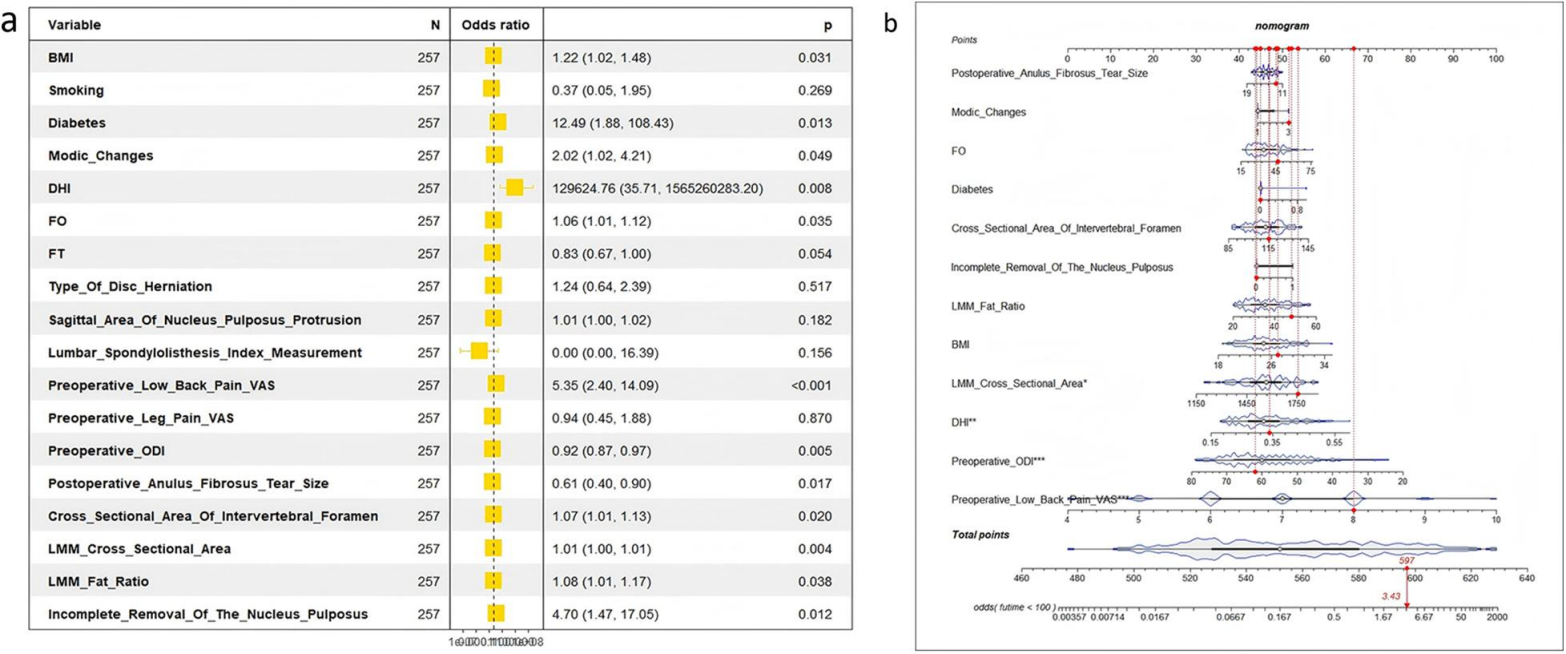
**(a)** forest plot of multi-factor logistic regression analysis; **(b)** nomogram of the uBED postoperative recurrence prediction model.

**Figure 7:**
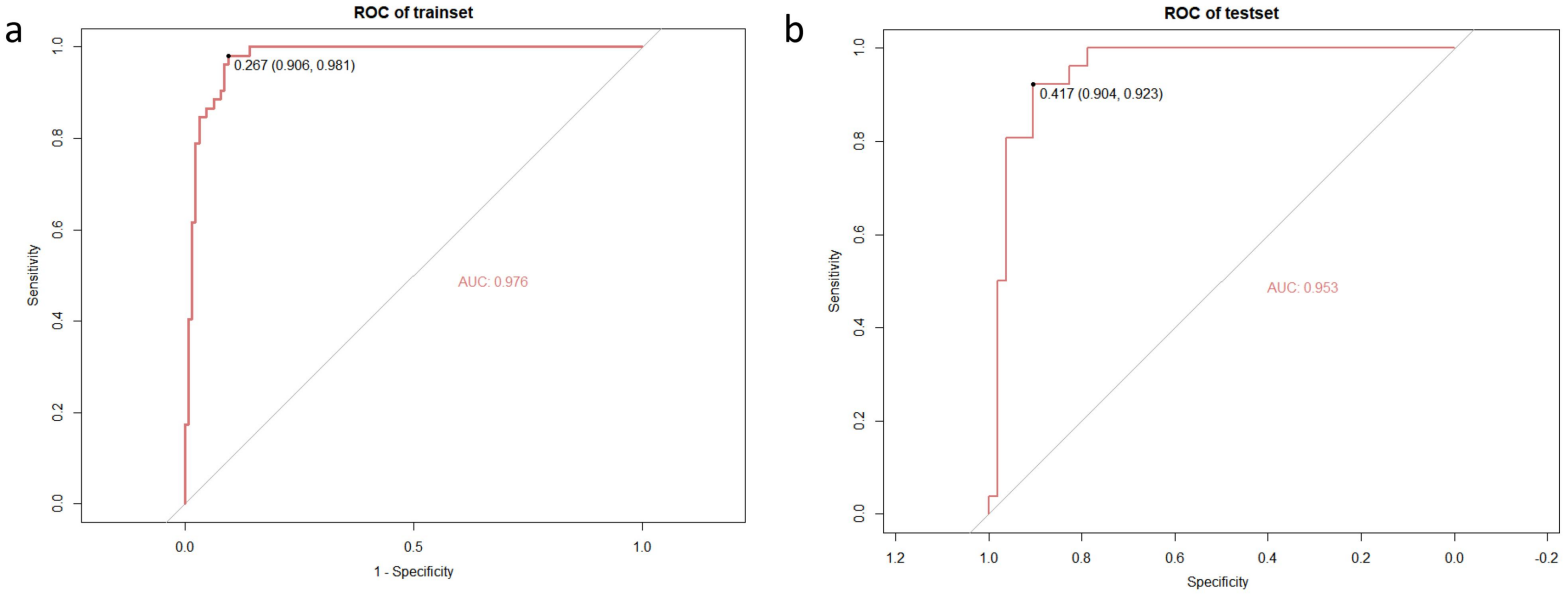
ROC curve of the postoperative recurrence prediction model for UBED; area under the curve (AUC) of the training group=0.976,area under the curve (AUC) of the validation group = 0.953.

**Figure 8:**
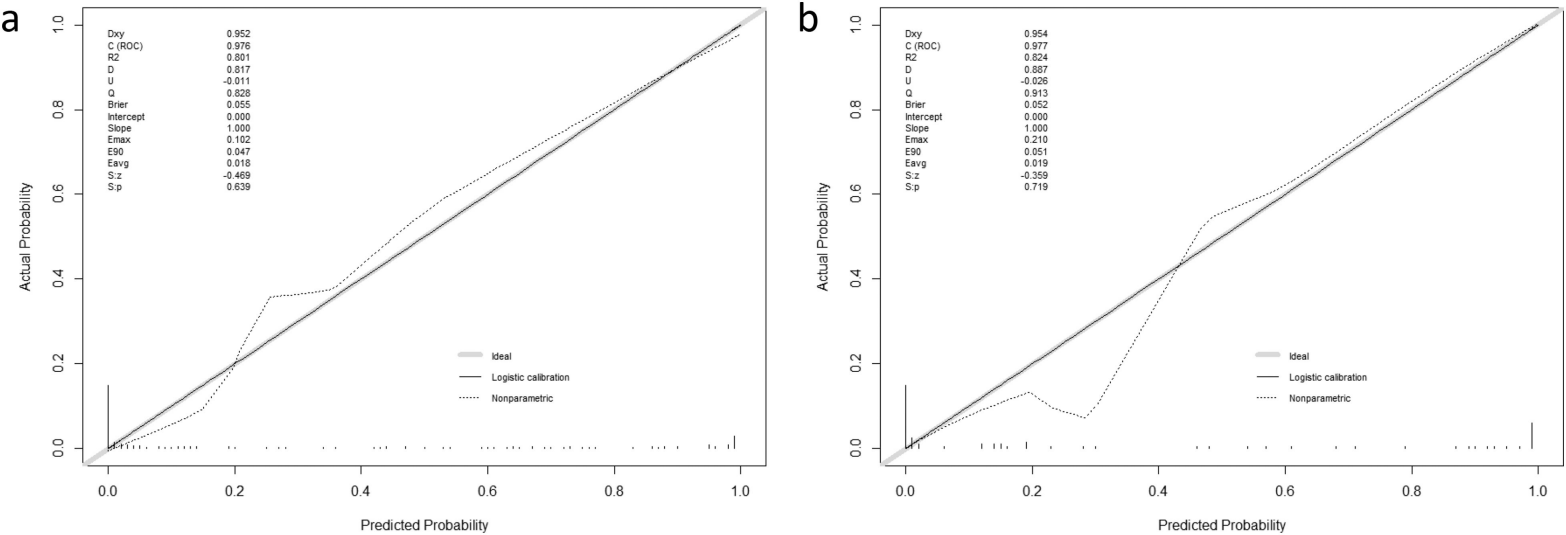
Calibration curve of the UBED postoperative recurrence prediction model,where bias-corrected is the calibration curve and the diagonal line ideal is the ideal curve; the corresponding P-value is S:p,and when S:*p* > 0.05,the calibration test is passed.

**Figure 9:**
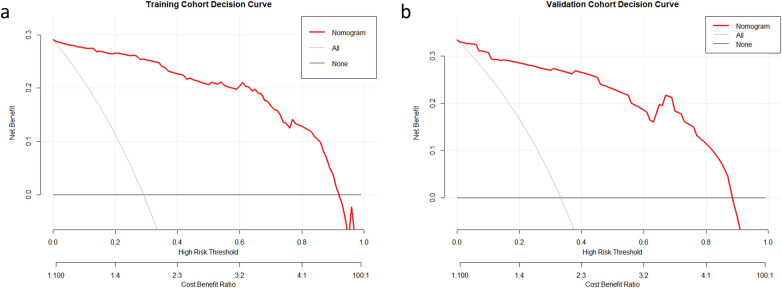
**(a)** is the training group, **(b)** is the validation group; Classifier represents the clinical diagnosis prediction model, ALL represents the assumption that all patients will experience disc recurrence,and None represents the assumption that none of the patients will experience recurrence.

A nomogram model was constructed based on multivariate logistic regression including the 18 selected variables. Results of the regression are presented as a forest plot in Figure 6a. Multivariate analysis identified 12 independent risk factors for postoperative recurrence after UBED: BMI, diabetes, surgical-level Modic changes, DHI, FO, preoperative low back pain VAS, preoperative ODI, postoperative annulus fibrosus tear size, CSAF, LMM cross-sectional area, LMM fat ratio, and incomplete nucleus pulposus removal. No significant multicollinearity was detected among these factors. A nomogram was developed using R, with points assigned to each risk factor. The total score, calculated by summing the points for all 12 indicators, corresponds to the predicted probability of postoperative recurrence on the bottom axis (Figure 6b). ROC curves were plotted using the pROC package. The AUC was 0.976 (95% CI: 0.906–0.981) in the training set (Figure 7a) and 0.953 (95% CI: 0.904–0.923) in the validation set (Figure 7b), indicating good discriminative ability of the model. Bootstrap resampling (1000 iterations) was used for internal and external validation to improve model stability. Calibration curves showed close alignment between the bias-corrected curve and the ideal diagonal, indicating good consistency between predicted and observed probabilities. The Hosmer-Lemeshow test yielded *P* = 0.164 for the training set (Figure 8a) and *P* = 0.481 for the validation set (Figure 8b), both >0.05, confirming satisfactory calibration. DCA was performed using the ggDCA package. The decision curves showed that the nomogram provided greater net benefit than the “treat all” or “treat none” strategies across threshold probabilities of 0–0.92 in the training set and 0–0.89 in the validation set (Figures 9a,b).

## 4 Discussion

Recurrent lumbar disc herniation (RLDH) is defined as re-herniation at the same level (ipsilateral or contralateral) causing nerve root compression and recurrent lumbocrural pain after a pain-free interval of 2 weeks to 6 months following primary discectomy (9). The incidence of RLDH ranges from 7% to 24%, associated with multiple risk factors. Depending on the clinical presentation, treatment options include revision endoscopic surgery or lumbar fusion (9). In our study, the recurrence rate was 9.34%, with a mean follow-up of more than 3 months. Most patients were manual workers (51%), and the remainder were mainly sedentary occupations such as teachers and civil servants. UBED preserves postoperative spinal stability, reduces wound contamination and infection risk, and accelerates recovery. Without the limitation of a rigid working cannula, it allows direct visualization for exploration, release, and extensive decompression of the spinal canal, achieving remarkable early therapeutic effects (18). Predicting recurrence after UBED can effectively prevent RLDH, avoid complications and revision surgery, and reduce healthcare costs. In this study, LASSO regression combined with multivariate logistic regression was used to screen risk factors for recurrence after UBED and construct a prediction model. Twelve predictive variables were finally identified: BMI, diabetes, surgical-level Modic changes, DHI, FO, preoperative low back pain VAS, preoperative ODI, postoperative annulus fibrosus tear size, CSAF, LMM cross-sectional area, LMM fat ratio, and incomplete nucleus pulposus removal. The nomogram model demonstrated good discrimination, with an AUC of 0.976 in the training set and 0.953 in the validation set. The Hosmer-Lemeshow test confirmed good calibration (*P* > 0.05 for both sets). DCA showed that the model yielded net clinical benefit across a wide range of threshold probabilities. Collectively, these results indicate that the model has robust discriminative and predictive performance in both datasets.

Interpretation of Individual Risk Factors Our model indicates that obesity increases the risk of RLDH. Multiple observational and retrospective studies have shown that higher BMI, particularly BMI≥30 kg/m^2^, is significantly associated with persistent symptoms, poor conservative treatment response, and higher recurrence rates after LDH (19, 20). Obesity may reduce physical activity, lower rehabilitation compliance and effectiveness, and increase spinal loading during exercise. We consider BMI control a key strategy for preventing LDH recurrence. Chronic hyperglycemia leads to the formation of advanced glycation end products (AGEs) and microvascular damage, accelerating disc degeneration and impairing repair capacity, thereby increasing recurrence risk (21, 22). Long-term glycemic control through diet, exercise, and pharmacotherapy can reduce AGE formation, delay microvascular disease, and alleviate nerve damage and chronic inflammation. Patients with Modic changes have higher recurrence rates than those without, attributed to persistent endplate inflammation and reduced biomechanical stability at the disc-endplate interface. Degenerative endplates continuously release inflammatory mediators such as cyclooxygenase-2, causing chronic low back pain and impaired functional recovery (23).

Reduced disc height is a hallmark of advanced disc degeneration. Uneven pressure distribution may cause residual or newly formed weak areas to re-herniate, mediated by deteriorated disc biomechanics and weakened annulus fibrosus (24, 25). Multiple retrospective studies and systematic reviews have confirmed that reduced preoperative disc height is an independent risk factor for recurrence (8, 26, 27). Lumbar facet joints work synergistically with the disc to maintain stability. Abnormal facet orientation causes uneven load distribution and increased shear stress, predisposing to annulus fibrosus tear and segmental instability, and increasing re-herniation risk (27).Facet joints restrict spinal rotation and excessive flexion-extension and share disc loading. Facet tropism (asymmetry in facet angle) leads to abnormal stress distribution and impaired anti-rotation capacity, increasing shear stress on the posterolateral annulus fibrosus during torsional movements (28). Vertebral spondylolisthesis subjects the adjacent disc to abnormal shear and rotational stress, accelerating degeneration. Studies show that disc herniation is more prevalent in patients with lumbar spondylolisthesis, typically occurring at the level above the slip (29). Higher spondylolisthesis grade (especially grade ≥ II) confers greater recurrence risk, driven by spinal instability. Fusion surgery to correct spondylolisthesis can reduce recurrence. High preoperative VAS scores have no direct causal link to recurrence but often indicate severe nerve compression, intense inflammation, or advanced disc degeneration. High preoperative ODI scores reflect greater functional limitation, frequently associated with widespread disc degeneration, nerve root adhesion, or large annulus fibrosus defects (17). The annulus fibrosus is avascular and nourished by diffusion from surrounding vessels. Tears >5 mm rarely achieve complete scar closure, and scar tissue formed over 3–6 months has reduced tensile strength. Tears >6 mm increase segmental loading by 30%, accelerating extrusion of residual nucleus pulposus. Persistent open tears allow intraspinal inflammatory factors (IL-6,TNF-*α*) to penetrate the disc, activate matrix metalloproteinases, and accelerate nucleus pulposus degradation, creating a vicious cycle of inflammation–degeneration–re-herniation (27, 30). CSAF is closely related to foraminal stenosis, and residual stenosis is a major cause of suboptimal symptom relief. Foraminal stenosis arises from facet hypertrophy, ligamentum flavum thickening, posterior vertebral osteophytes, segmental instability, facet degeneration, and disc height loss. These factors increase abnormal stress on the affected segment, accelerate disc degeneration, and elevate the risk of annulus re-tear and re-herniation (31). Patients with LDH frequently develop disuse atrophy of the multifidus muscle due to pain, nerve compression, and reduced activity. Decreased cross-sectional area and muscle strength impair lumbar stability. Studies demonstrate that multifidus atrophy impairs control of excessive flexion, rotation, and shear, leading to segmental instability (32). Unstable segments subject the disc to greater and uneven mechanical loads. Altered multifidus biomechanics also affect lumbar curvature and intervertebral stress, damaging the healing annulus and nucleus pulposus, and cumulative microtrauma ultimately leads to recurrence. Multifidus fat infiltration significantly increases recurrence risk after percutaneous endoscopic discectomy by disrupting spinal stability, inducing chronic inflammation, and reducing muscle mechanical performance. Patients with severe fat infiltration (≥25%) have a 5.5-fold higher recurrence rate and 3.4-fold higher independent risk than those with mild infiltration. Preoperative assessment of multifidus fat infiltration is clinically valuable for predicting recurrence, optimizing surgical planning, and designing individualized rehabilitation (33). The degree of multifidus atrophy (reduced cross-sectional area and fat infiltration) correlates positively with LDH severity, functional impairment, and recurrence risk (34). Moderate to severe fat infiltration weakens dynamic support for the operated segment, worsens postoperative local biomechanics, and predisposes to ipsilateral same-level recurrence. Rehabilitation strategies targeting multifidus muscle recovery (reducing fat infiltration, improving strength and endurance) are essential for preventing recurrence. A 25% fat infiltration threshold is commonly used clinically to define severe involvement (33, 35). Severe fat infiltration (FI ≥ 25%) is associated with a 12.6% recurrence rate versus 2.3% for mild infiltration (OR = 3.41). Multifidus fat infiltration drives spinal biomechanical imbalance, inflammatory microenvironment changes, and neuromuscular dysfunction. Disc degeneration and fat infiltration form an inflammation–degeneration cycle: degenerated discs release inflammatory mediators that promote muscle fatty infiltration, which further reduces stability. One study reported that patients with severe multifidus fat infiltration had 45% higher ODI scores and persistently worse back pain VAS scores at 12 months postoperatively (33). Incomplete nucleus pulposus removal allows residual disc tissue to extrude under persistent spinal loading, typically through the damaged or weakened annulus fibrosus. Re-contact with the nerve root triggers inflammation and recurrent radicular pain (36). Other studies have identified additional risk factors including age >60 years, small FO angle, large FT, high DHI, large sROM, narrow herniation base, Scheuermann's disease, mild index-level disc degeneration, severe adjacent-level degeneration, protrusion-type herniation, lumbosacral transitional vertebra, and surgeon inexperience (9, 37, 38). Wang et al. described a learning curve for interlaminar endoscopic surgery: operative time decreases significantly with accumulating case volume (39). Bleeding obscures the surgical field, and unfamiliarity with endoscopic anatomy can iatrogenically damage the annulus fibrosus or enlarge existing tears, predisposing to recurrence (40).

This study has several limitations. First, it is a single-center retrospective study with a relatively small sample size and limited recurrence events, which may introduce selection bias. Results may be influenced by the local patient population, surgeon experience, and postoperative care protocols at our center, limiting generalizability to other institutions.

Second, all radiological measurements were performed with computer-assisted tools, and minor resolution differences in x-ray, CT, and MRI, as well as variations in patient positioning and image rotation, may introduce measurement error and inflate type I error from multiple comparisons.

Third, the patient cohort was limited to the Panyu district of Guangzhou. Multicenter studies across broader geographic regions are needed. The high number of predictors relative to recurrence events (EPV ≈ 2:1) creates a substantial risk of overfitting.

Fourth, follow-up duration was relatively short. Recurrence can occur months to years postoperatively, and longer follow-up would better capture late recurrences and associated risk factors. Loss to follow-up may also have underestimated the true recurrence rate.

Fifth, this study did not include a comparison group of patients treated with other surgical approaches (e.g., open surgery, single-channel endoscopy), so it cannot determine whether UBED has unique recurrence risk factors.

Finally, not all patients underwent postoperative MRI follow-up. The definition of “incomplete nucleus pulposus removal” relied on subjective surgical record documentation, lacking objective verification by intraoperative ultrasound or immediate postoperative MRI. This may introduce measurement bias inherent to retrospective design. Future prospective studies should adopt standardized, imaging-based definitions of residual nucleus pulposus to validate our findings.

With only 24 recurrence events among 257 patients and an EPV of approximately 2:1, this study falls far short of the recommended EPV ≥ 10:1 for prediction model development. The validation set contained only about 7 events, which is insufficient to reliably validate a 12-predictor model. This is the primary limitation of the study: the model carries a high risk of overfitting, performance metrics are likely optimistically biased, and external generalizability is limited.

In summary, the nomogram prediction model for recurrence after UBED developed in this study demonstrates good discrimination and calibration in both training and validation sets. However, limited by small sample size, few recurrence events, low EPV, and insufficient validation events, the model has notable overfitting risk, optimistic performance estimates, and limited external applicability.

This model should be regarded as an exploratory clinical prediction tool, suitable for assisting spine surgeons in identifying patients at high recurrence risk, facilitating preoperative counseling, and guiding intervention on modifiable factors (e.g., weight management, glycemic control, smoking cessation, multifidus muscle rehabilitation). It cannot replace comprehensive clinical assessment and decision-making. Future multicenter, large-sample, prospective cohort studies with long-term follow-up are warranted to further validate and optimize the model and evaluate its real-world clinical utility.

## Data Availability

The raw data supporting the conclusions of this article will be made available by the authors, without undue reservation.
